# Serving With Purpose: How Servant Leadership Fosters Nurses’ Service Orientation and Performance

**DOI:** 10.1155/jonm/4577566

**Published:** 2026-10-09

**Authors:** Lihsiang Shih, Fenghuang Yang, Lifen Wang

**Affiliations:** ^1^ Department of Nursing Cheng Ching General Hospital Taichung Taiwan ccgh.com.tw; ^2^ Department of Nursing Chung Kang Branch Cheng Ching Hospital Taichung Taiwan ccgh.com.tw; ^3^ Master’s Degree Program in Long-Term Care Central Taiwan University of Science and Technology Taichung Taiwan ctust.edu.tw

**Keywords:** nursing management, servant leadership, service orientation, service performance, service rule commitment

## Abstract

**Background:**

Servant leadership emphasizes humility, ethical conduct, service to others, and concern for followers’ development. In nursing teams, understanding how servant leadership is associated with nurses’ service performance and the mechanisms underlying this association is important for patient‐centered service delivery and nursing management.

**Aim:**

To examine the association between team‐level servant leadership and individual nurses’ service performance and to test two proposed indirect pathways through team service orientation and individual service rule commitment.

**Methods:**

A multilevel, time‐lagged, and multisource survey was conducted across 44 nursing teams from regional teaching hospitals in Taiwan. Data were collected at two time points, 4 weeks apart, using matched questionnaires completed by nurses and their immediate supervisors. A total of 193 valid matched responses were obtained. Nurses assessed servant leadership, service orientation, and service rule commitment, whereas supervisors rated nurses’ service performance. Random‐intercept multilevel models were estimated using full maximum likelihood. The service orientation pathway was specified as a 2‐2‐1 model and the service rule commitment pathway as a 2‐1‐1 model. Formal indirect effects were evaluated using Monte Carlo confidence intervals.

**Results:**

Servant leadership was positively associated with nurses’ service performance before the proposed mediators were entered (*B* = 0.230, SE = 0.112, *p* = 0.046). Servant leadership was also positively associated with team service orientation (*B* = 0.428, SE = 0.105, *p* < 0.001), which in turn was positively associated with nurses’ service performance (*B* = 0.371, SE = 0.149, *p* = 0.017). The indirect effect through team service orientation was statistically significant (effect = 0.159, Monte Carlo SE = 0.077, 95% CI [0.030, 0.328]). In contrast, the indirect effect through service rule commitment was not statistically significant (effect = −0.027, Monte Carlo SE = 0.038, 95% CI [−0.115, 0.038]).

**Conclusion:**

Team‐level servant leadership was positively associated with nurses’ service performance and team service orientation. The statistically significant indirect effect through team service orientation supports a value‐based indirect pathway linking servant leadership to nurses’ service performance, whereas the proposed rule‐based indirect pathway through service rule commitment was not supported. These findings suggest that servant leadership may be more closely associated with the internalization of patient‐centered service values than with additional commitment to formal service rules.

**Implications for Nursing Management:**

Nurse managers may cultivate service‐oriented team environments by modeling patient‐centered values, demonstrating humility and ethical responsibility, providing supportive feedback, and promoting staff development. Leadership development programs should complement formal service standards with value‐based practices that help nurses connect organizational expectations with professional meaning and patient‐centered care.

## 1. Introduction

Nurses play a central role in maintaining patient safety, continuity of care, and service quality. However, healthcare systems are facing increasing pressure from workforce shortages, rising patient complexity, and growing expectations for patient‐centered care. The World Health Organization [1] estimated a global shortage of nearly six million nurses by 2030, highlighting the need for organizational strategies that support nurses in delivering high‐quality care. In this context, improving nurses’ service performance is an important concern for nursing management because it reflects how nurses translate professional competence, responsiveness, and patient‐centered values into everyday care.

Leadership is a key organizational factor in sustaining nursing performance and care quality. Among different leadership approaches, servant leadership has received growing attention in healthcare because it emphasizes humility, service to others, ethical responsibility, and concern for followers’ growth. These characteristics are particularly relevant in nursing teams, where care delivery depends not only on technical competence but also on compassion, responsiveness, and professional commitment. However, rather than assuming that servant leadership is inherently aligned with nursing values, it is necessary to examine how such leadership behaviors are associated with nurses’ service‐related attitudes and performance.

Servant leadership, first articulated by Greenleaf [2, 3], is grounded in the principle that leaders prioritize the needs, development, and well‐being of followers while fostering a broader culture of service. Validated measures such as the SL‐28 and its short form, the SL‐7, have enabled empirical investigation of servant leadership across organizational settings [4, 5]. Prior research has linked servant leadership to trust, psychological safety, organizational citizenship behavior, and performance [6–8].

Evidence from healthcare settings further suggests that servant leadership may be beneficial for nursing work environments and staff outcomes. A recent systematic review of servant leadership in healthcare found that servant leadership has been examined in relation to a range of individual and organizational outcomes and may contribute to workforce commitment and performance excellence in healthcare [9]. Recent healthcare studies and reviews also indicate that servant leadership is associated with favorable nursing work environments, professional attitudes, and staff outcomes among nurses [10–12]. In the nursing context, Xiao et al. [13] conceptualized servant leadership as a social support resource that prioritizes followers’ growth and development and can provide emotional support, constructive feedback, useful information, and recognition. Saleem et al. [14] further reported that servant leadership contributed to public hospital performance through trust in leaders and psychological empowerment among nurses. Together, these findings establish the relevance of servant leadership in nursing, but the pathways linking servant leadership to nurses’ service performance remain unclear.

Research in service‐oriented industries also provides useful insights into the performance implications of servant leadership. Chen et al. [15] showed that servant leadership was associated with frontline service performance through self‐identity and group identification. Chiniara and Bentein [16] demonstrated that servant leaders supported employees’ autonomy, competence, and relatedness, which in turn enhanced performance‐related outcomes. A meta‐analysis by Lee et al. [17] further indicated that servant leadership explains incremental variance in outcomes beyond other positive leadership styles. Collectively, these studies suggest that servant leadership may shape performance through motivational, relational, and identity‐based mechanisms.

Despite this growing evidence, several gaps remain. First, much of the servant leadership literature has focused on attitudinal outcomes, such as job satisfaction, trust, and well‐being, whereas fewer studies have examined service performance. In nursing, service performance is especially important because it reflects both technical proficiency and relational care behaviors, including empathy, responsiveness, reliability, and sensitivity to patients’ needs. These behaviors are directly relevant to patient safety, care continuity, and patient‐centered service quality.

Second, the pathways linking servant leadership to service performance remain insufficiently understood. One potential pathway is value‐based. Servant leaders may strengthen service orientation, defined as a predisposition to provide attentive, responsive, and high‐quality service [18]. A second potential pathway is rule‐based, involving service rule commitment, or the extent to which employees are committed to organizational service standards and expectations [19]. These pathways represent conceptually distinct forms of service‐related motivation: Service orientation reflects the internalization of service values, whereas service rule commitment reflects commitment to formal service expectations. Distinguishing between these pathways is particularly relevant to nursing management, where effective service delivery requires both professional values and adherence to organizational standards. Third, important methodological limitations remain in existing literature. Many previous studies have relied on cross‐sectional, single‐source, or single‐level designs, which raise concerns about common method bias and provide limited insight into cross‐level leadership processes. Because nursing care is delivered within hierarchical and team‐based systems, multilevel, multisource, and time‐lagged designs are needed to examine how team‐level servant leadership is associated with individual nurses’ service performance.

The present study draws on social learning theory and social exchange theory to explain the two proposed pathways. Social learning theory proposes that individuals acquire values and behaviors by observing salient role models [20]. Within nursing teams, servant leaders may provide behavioral cues through humility, ethical conduct, respect, and patient‐centered actions. Repeated exposure to these behaviors may help establish a shared orientation toward attentive and responsive service, providing a theoretical basis for the proposed pathway through team service orientation. Social exchange theory provides a complementary explanation. When nurses perceive that leaders demonstrate care, respect, and support, they may reciprocate through stronger commitment and improved service performance [21, 22]. Nevertheless, servant leadership primarily emphasizes follower development, support, and value‐based influence rather than direct rule enforcement. Its association with service rule commitment may therefore be less pronounced than its association with service orientation. Accordingly, this study examines two theoretically distinct multilevel indirect pathways linking team‐level servant leadership to individual nurses’ service performance: A value‐based pathway through team service orientation and a rule‐based pathway through service rule commitment. By examining these pathways simultaneously, the study seeks to clarify whether servant leadership is more closely associated with shared service values or with commitment to formal service standards in nursing teams.

This study contributes to nursing management research in three ways. First, it extends servant leadership research by focusing on service performance, an outcome with direct clinical and managerial relevance. Second, it distinguishes between service orientation and service rule commitment as two theoretically different explanatory pathways linking servant leadership to service performance. Third, it addresses methodological limitations of prior research by using a multilevel, time‐lagged and multisource design that reflects the nested structure of nursing teams.

### 1.1. Aims

This study aimed to:

1. Examine the association between team‐level servant leadership and individual nurses’ service performance in hospital nursing teams.
2. Investigate whether service orientation and service rule commitment mediate this association. Test two proposed multilevel indirect pathways linking servant leadership to nurses’ service performance through team service orientation and service rule commitment.

## 2. Methods

### 2.1. Theoretical Framework

This study was guided by social learning theory [20] and social exchange theory [21, 22] to explain the pathways through which servant leadership may be associated with nurses’ service performance. Social learning theory suggests that individuals acquire values and behaviors by observing salient role models. In nursing teams, servant leaders who demonstrate humility, empathy, ethical responsibility, and patient‐centered conduct may provide behavioral cues that strengthen nurses’ service orientation. Accordingly, this theory supports the hypothesized pathway from servant leadership to service orientation and, subsequently, to service performance. Social exchange theory emphasizes reciprocity in leader–follower relationships. When nurses perceive that servant leaders provide genuine care, respect, and support, they may reciprocate through stronger commitment to organizational expectations and improved service performance. This theory therefore supports the hypothesized pathway from servant leadership to service rule commitment and, subsequently, to service performance. Based on these theoretical perspectives, the present study examined a multilevel conceptual model in which team‐level servant leadership was linked to individual nurses’ service performance through two mechanisms: service orientation as a value‐based mechanism and service rule commitment as a rule‐based mechanism.

### 2.2. Study Design

A multilevel, time‐lagged, and multisource survey design was employed to test the hypothesized model. Given the hierarchical structure of healthcare organizations, hierarchical linear modeling (HLM) was used to examine how team‐level servant leadership was associated with individual nurses’ service performance through team‐level service orientation and individual‐level service rule commitment. Three data sources were used: Nurses’ self‐reports of servant leadership, service orientation, and service rule commitment; supervisors’ ratings of nurses’ service performance; and demographic and organizational information from administrators. Data were collected at two time points separated by 4 weeks. This interval was selected to provide temporal separation between nurses’ reports of leadership and service‐related attitudes and supervisors’ subsequent ratings of service performance, thereby helping to reduce common method bias. The 4‐week interval was also considered practical and feasible in busy clinical settings and helped maintain participant retention across the two waves of data collection.

### 2.3. Setting and Sample

This study was conducted in nursing units of regional teaching hospitals in Taiwan using convenience sampling. The study population consisted of frontline registered nurses and their immediate head nurses. Eligible nurses were licensed registered nurses engaged in direct patient care, whereas eligible head nurses had served in their managerial role for at least 6 months to ensure sufficient familiarity with unit members and stable leader–subordinate interactions. Only nurse–head nurse dyads with complete matched data were included. Nurses who were temporary or contract staff, students, trainees, or individuals holding leadership positions beyond the immediate unit level were excluded. A total of 215 nurse–head nurse dyads were invited to participate. After excluding incomplete or unmatched responses, 193 valid dyads were obtained, yielding an effective response rate of 89.7%. The final sample included 44 nursing units, with an average team size of 17 members. Among participating nurses, 96.4% were female, 29.5% were younger than 25 years, 28.5% had more than 10 years of clinical experience, and 54.4% held a bachelor’s degree.

### 2.4. Data Collection

Organizational consent was obtained from each participating hospital before data collection. At Time 1, staff nurses completed questionnaires assessing servant leadership, service orientation, and service rule commitment. At Time 2, 4 weeks later, nurse supervisors independently rated the corresponding nurses’ service performance. To ensure confidentiality, completed questionnaires were sealed in envelopes and returned to designated hospital contacts, who then forwarded them to the research team. No identifying information was attached to the survey data, and matched responses were handled using coded identifiers known only to the research team.

### 2.5. Measurement

All study instruments originally developed in English were translated into Chinese using a back‐translation procedure [23]. Following translation, the measures were reviewed by experts for semantic equivalence and contextual appropriateness, and item wording was refined where necessary to ensure relevance to the healthcare setting. In particular, customer‐related wording in the service orientation and service performance measures was adapted to refer to patients and healthcare services while preserving the substantive meaning of the original items. No separate pilot testing was conducted. Servant leadership and service orientation were assessed using 7‐point Likert scales, whereas service rule commitment and service performance were assessed using 5‐point Likert scales. Higher scores indicated higher levels of the corresponding constructs. Internal consistency was evaluated using Cronbach’s *α*.

#### 2.5.1. Demographic Characteristics

Demographic characteristics were collected to describe the sample and control for potential confounding influences. At the individual level, variables included gender, age, education level, total length of nursing service, and tenure with the immediate supervisor. At the team level, unit size was documented to reflect structural variation across nursing units.

#### 2.5.2. Servant Leadership

Servant leadership was measured using the SL‐7 short scale developed by Liden et al. [5], which was derived from the original SL‐28 [4]. The scale consists of seven items, each reflecting one dimension of servant leadership, such as putting subordinates first, emotional healing, and moral integrity. A sample item was, “My leader considers my career development as a priority.” Items were rated on a 7‐point Likert scale ranging from 1 (*strongly disagree*) to 7 (*strongly agree*), with higher scores indicating stronger perceptions of servant leadership. Cronbach’s *α* in this study was 0.90.

#### 2.5.3. Service Orientation

Service orientation was assessed using the five‐item scale developed by Bettencourt et al. [18], adapted from Van Dyne et al. [24]. The scale evaluates the extent to which nurses demonstrate patient‐centered service attitudes. A representative item was, “I am proud to provide thoughtful service to patients.” Items were rated on a 7‐point Likert scale ranging from 1 (*strongly disagree*) to 7 (*strongly agree*), with higher scores indicating stronger service orientation. Cronbach’s *α* in this study was 0.86.

#### 2.5.4. Service Rule Commitment

Service rule commitment was measured using the five‐item scale developed by Gosserand and Diefendorff [19]. This scale assesses nurses’ commitment to organizational service rules and standards. An example item was, “I provide patient care strictly in accordance with hospital service rules.” Items were rated on a 5‐point Likert scale ranging from 1 (*strongly disagree*) to 5 (*strongly agree*), with higher scores indicating stronger service rule commitment. Cronbach’s *α* in this study was 0.79.

#### 2.5.5. Service Performance

Service performance was rated by nurse supervisors using the 20‐item scale developed by Liao et al. [25], adapted from Parasuraman et al. [26]. The scale captures the quality and consistency of service behaviors. A sample item was, “This nurse reliably delivers medical services as promised.” Items were rated on a 5‐point Likert scale ranging from 1 (*strongly disagree*) to 5 (*strongly agree*), with higher scores indicating better service performance. Cronbach’s α in this study was 0.97. Given the high internal consistency of the scale, additional item‐level diagnostics were conducted to examine potential item redundancy. Corrected item‐total correlations ranged from 0.657 to 0.870, and Cronbach’s α if an item was deleted ranged from 0.971 to 0.973. Although some item pairs were strongly correlated, deletion of individual items did not meaningfully improve reliability. Accordingly, all 20 items were retained. No item parceling was used in the confirmatory factor analysis (CFA); all 20 service performance items were modeled individually.

#### 2.5.6. Control Variables

Several control variables were included to account for potential confounding factors. At the individual level, nurse age and tenure with the immediate supervisor, as well as power distance, were controlled. Age and tenure with the immediate supervisor were included because previous research suggests that these characteristics may be associated with employee attitudes, perceptions of leadership, and performance‐related outcomes [27, 28]. Power distance was measured using the six‐item individual‐level power distance orientation scale developed by Dorfman and Howell [29]. A sample item was, “When making decisions, supervisors do not need to consult their subordinates.” Items were rated on a 5‐point Likert scale ranging from 1 (*strongly disagree*) to 5 (*strongly agree*), with higher scores indicating greater acceptance of hierarchical power differences. Cronbach’s *α* was 0.79 in the present study. At the team level, unit size was included as a control variable.

### 2.6. Data Analysis

Data were analyzed using SPSS 26.0, HLM 7.0, and LISREL 8.7. Descriptive statistics were used to summarize sample characteristics, and Pearson’s correlation was used to examine bivariate associations among the study variables. CFA was conducted in LISREL 8.7 to evaluate the four‐factor measurement model comprising servant leadership, service orientation, service rule commitment, and service performance. Model fit was assessed using the comparative fit index (CFI), normed fit index (NFI), non‐normed fit index (NNFI), root mean square error of approximation (RMSEA), and standardized root mean square residual (SRMR). Because 193 nurses were nested within 44 nursing teams, HLM was used to examine the cross‐level relationships [30, 31]. Servant leadership and service orientation were specified at the team level (Level 2), whereas service rule commitment and service performance were specified at the individual level (Level 1). Aggregation of servant leadership and service orientation to the team level was evaluated using *r*wg, intraclass correlation coefficient (ICC) (1), and ICC (2) [32, 33]. Age, tenure with the immediate supervisor, and power distance were grand‐mean‐centered at Level 1. Team size, servant leadership, and team service orientation were grand‐mean‐centered at Level 2. For the 2‐1‐1 pathway, service rule commitment was partitioned into a group‐mean‐centered within‐team component and a grand‐mean‐centered team‐mean component to separate within‐team and between‐team effects [34]. Random‐intercept models were estimated using full maximum likelihood. The service orientation pathway was specified as a 2‐2‐1 model, whereas the service rule commitment pathway was specified as a 2‐1‐1 model [35]. For the 2‐2‐1 pathway, the association between servant leadership and team service orientation was estimated at Level 2 using ordinary least‐squares regression across the 44 teams, controlling for team size. Indirect effects were evaluated using Monte Carlo confidence intervals based on the unstandardized path coefficients and their standard errors [36]. An indirect effect was considered statistically significant when the 95% confidence interval did not include zero. All tests were two‐tailed, with *p* < 0.05 indicating statistical significance.

### 2.7. Ethical Considerations

The study was conducted in accordance with the Declaration of Helsinki. Ethical approval was obtained from the Institutional Review Board of Kuang Tien General Hospital (Approval No. 10441). Written informed consent was obtained from all participants before participation. Participation was voluntary, and respondents were informed of their right to withdraw at any stage without penalty. Confidentiality was strictly maintained. Data were anonymized, consent forms were stored separately from questionnaires, and access was restricted to the research team. No personal or identifying information was disclosed in any publications.

## 3. Results

### 3.1. Sample Characteristics

Among the 193 valid individual‐level responses, most nurses were female (96.4%), whereas males accounted for 3.6%. In terms of age, 29.5% were younger than 25 years, 23.3% were aged 26–30 years, 22.8% were aged 31–35 years, 13.5% were aged 36–40 years, and 10.9% were older than 40 years. Regarding work experience, 28.5% had more than 10 years of nursing service, 23.8% had 5–10 years, 16.1% had 1–3 years, 15.5% had less than one year, and 10.9% had 3–5 years. Regarding educational attainment, 54.4% held a bachelor’s degree, 41.5% held a diploma, 2.1% reported a postgraduate degree, and 1.6% had a high school education or below. At the team level, the sample comprised 44 nursing teams, with an average team size of 17.23 members (SD = 10.60). Supervisors were predominantly head nurses and had an average of 12.3 years of leadership experience.

### 3.2. Correlation Analysis

The means, standard deviations, and correlations of the study variables are presented in Table 1. At the individual level, nurse age was positively correlated with tenure with supervisor (*r* = 0.49, *p* < 0.001), power distance (*r* = 0.20, *p* < 0.01), service rule commitment (*r* = 0.18, *p* < 0.05), and service performance (*r* = 0.17, *p* < 0.05). Tenure with supervisor was positively correlated with service performance (*r* = 0.27, *p* < 0.001). Service rule commitment was not significantly correlated with service performance (*r* = 0.02, *p* > 0.05). At the team level, servant leadership was positively correlated with service orientation (*r* = 0.52, *p* < 0.05). Team size was not significantly correlated with servant leadership or service orientation. None of the correlation coefficients exceeded 0.80, suggesting that multicollinearity was unlikely to be a serious concern in subsequent analyses.

**TABLE 1:** Descriptive statistics and correlation matrix.

Level‐1 individual level (*N* = 193)						
Variables	M	SD	1	2	3	4
1. Age	30.97	7.56				
2. Tenure with supervisor (years)	3.60	3.75	0.49^∗∗∗^			
3. Power distance	2.19	0.59	0.20^∗∗^	0.05		
4. Service rule commitment	3.66	0.61	0.18^∗^	0.13	−0.10	
5. Service performance	3.75	0.66	0.17^∗^	0.27^∗∗∗^	0.14	0.02

**Level-2 team level (*N* = 44)**						
**Variables**	**M**	**SD**	**1**	**2**		

1. Team size	17.23	10.60				
2. Servant leadership	4.99	0.59	−0.12			
3. Service orientation	5.41	0.47	0.10	0.52^∗^		

*Note:* Tenure with supervisor measured in years.

Abbreviations: M, mean; SD, standard deviation.

^∗^ *p* < 0.05.

^∗∗^ *p* < 0.01.

^∗∗∗^ *p* < 0.001.

### 3.3. CFA

Before testing the hypotheses, CFA was conducted using LISREL 8.7 to assess the measurement properties of servant leadership, service orientation, service rule commitment, and service performance. The hypothesized four‐factor model demonstrated acceptable fit to the data (*χ* ^2^ = 1408.97, df = 623, *χ* ^2^/df = 2.26, CFI = 0.97, NFI = 0.94, NNFI = 0.96, RMSEA = 0.08, and SRMR = 0.06). Standardized factor loadings ranged from 0.60 to 0.89 for servant leadership, 0.67 to 0.83 for service orientation, 0.50 to 0.81 for service rule commitment, and 0.65 to 0.88 for service performance. Composite reliability values were 0.90, 0.86, 0.81, and 0.97, respectively, and the corresponding AVE values were 0.57, 0.56, 0.46, and 0.65. To further assess construct distinctiveness, the hypothesized four‐factor model was compared with three alternative three‐factor models. The first alternative model combined servant leadership and service orientation (*χ* ^2^ = 1996.09, df = 626, RMSEA = 0.11, CFI = 0.95, NFI = 0.92, NNFI = 0.94). The second combined servant leadership and service rule commitment (*χ* ^2^ = 1760.98, df = 626, RMSEA = 0.10, CFI = 0.95, NFI = 0.92, NNFI = 0.95), whereas the third combined servant leadership, service orientation, and service rule commitment into a single factor (*χ* ^2^ = 1773.36, df = 626, RMSEA = 0.10, CFI = 0.95, NFI = 0.92, NNFI = 0.95). The hypothesized four‐factor model provided a significantly better fit than all three alternative models. Discriminant validity was further supported because the confidence intervals for correlations between all pairs of latent constructs did not include 1.00.

### 3.4. Hypothesis Testing

#### 3.4.1. Multilevel Models Predicting Nurses’ Service Performance

The null model for service performance yielded a between‐team variance component of 0.146 and a Level‐1 residual variance of 0.266, corresponding to an ICC of 0.354. In the control model (Model 1), the addition of age, tenure with the immediate supervisor, power distance, and team size significantly improved model fit relative to the null model (Δdeviance = 10.30, Δdf = 4, *p* = 0.036). Adding servant leadership in Model 2 resulted in a further improvement in model fit (Δdeviance = 4.08, Δdf = 1, *p* = 0.043), and servant leadership was positively associated with nurses’ service performance (*B* = 0.230, SE = 0.112, *t* = 2.06, *p* = 0.046). When team service orientation was added in Model 3, model fit improved significantly (Δdeviance = 5.82, Δdf = 1, *p* = 0.016). Team service orientation was positively associated with nurses’ service performance (*B* = 0.371, SE = 0.149, *t* = 2.50, *p* = 0.017), whereas the coefficient for servant leadership decreased and was no longer statistically significant (*B* = 0.079, SE = 0.121, *p* = 0.518). In Model 4, neither the group‐mean‐centered within‐team component of service rule commitment (*B* = −0.033, SE = 0.076, *p* = 0.669) nor the grand‐mean‐centered team‐mean component (*B* = −0.151, SE = 0.176, *p* = 0.397) was significantly associated with nurses’ service performance. The addition of these two components did not improve model fit (Δdeviance = 0.91, Δdf = 2, *p* = 0.635). Full model estimates are presented in Table 2.

**TABLE 2:** Multilevel models predicting nurses’ service performance.

Variables	Nurses’ service performance				
	Model 0	Model 1	Model 2	Model 3	Model 4
Intercept	3.796^∗∗∗^ (0.069)	3.793^∗∗∗^ (0.065)	3.793^∗∗∗^ (0.062)	3.794^∗∗∗^ (0.058)	3.793^∗∗∗^ (0.058)
Level 1					
Tenure with supervisor	—	0.024 (0.013)	0.020 (0.013)	0.018 (0.013)	0.018 (0.013)
Age	—	0.007 (0.007)	0.008 (0.007)	0.007 (0.006)	0.008 (0.007)
Power distance	—	0.091 (0.072)	0.093 (0.071)	0.102 (0.071)	0.096 (0.072)
Service rule commitment (within‐team)	—	—	—	—	−0.033 (0.076)
Level 2					
Team size	—	0.003 (0.006)	0.005 (0.006)	0.002 (0.006)	0.002 (0.006)
Servant leadership	—	—	0.230^∗^ (0.112)	0.079 (0.121)	0.099 (0.123)
Service orientation	—	—	—	0.371^∗^ (0.149)	0.396^∗^ (0.151)
Service rule commitment (team mean)	—	—	—	—	−0.151 (0.176)
Model statistics					
Random‐intercept variance, *τ*_00_	0.146	0.122	0.109	0.088	0.087
Level‐1 residual variance, *σ* ^2^	0.266	0.258	0.257	0.256	0.256
Pseudo‐*R* ^2^, Level 1	—	0.030	0.035	0.036	0.039
Pseudo‐*R* ^2^, Level 2	—	0.162	0.255	0.393	0.403
Deviance	343.07	332.77	328.69	322.87	321.96

*Note:* Unstandardized coefficients are reported, with standard errors in parentheses. Model 0 is the unconditional model; Model 1 includes controls; Model 2 adds servant leadership; Model 3 adds team service orientation; and Model 4 adds the within‐team and team‐mean components of service rule commitment. Level‐1 controls and Level‐2 predictors were grand‐mean‐centered; the within‐team component of service rule commitment was group‐mean‐centered. Models were estimated using full maximum likelihood with random intercepts. *N* = 193 nurses nested within 44 teams.

^∗^ *p* < 0.05.

^∗∗∗^ *p* < 0.001.

#### 3.4.2. Models Predicting Service Orientation and Service Rule Commitment

At the team level, servant leadership was positively associated with team service orientation (*B* = 0.428, SE = 0.105, t = 4.064, *p* < 0.001, 95% CI [0.215, 0.640]). The model explained 29.5% of the variance in team service orientation (adjusted *R* ^2^ = 0.260). For service rule commitment, the null model yielded an ICC of 0.138. The control model significantly improved model fit relative to the null model (Δdeviance = 12.35, Δdf = 4, *p* = 0.015). Adding servant leadership did not significantly improve model fit (Δdeviance = 3.57, Δdf = 1, *p* = 0.059). Servant leadership was positively but not significantly associated with service rule commitment (*B* = 0.178, *SE* = 0.092, *t* = 1.93, *p* = 0.060). Age was positively associated with service rule commitment (*B* = 0.015, *SE* = 0.007, *p* = 0.020), whereas power distance was negatively associated with service rule commitment (*B* = −0.170, *SE* = 0.072, *p* = 0.020). Full multilevel estimates are presented in Table 3.

**TABLE 3:** Models predicting service orientation and service rule commitment.

Variables	Service orientation		Service rule commitment	
	Team‐level model	Model 0	Model 1	Model 2
Service orientation model				
Intercept	3.151^∗∗∗^ (0.549)			
Team size	0.007 (0.006)			
Servant leadership	0.428^∗∗∗^ (0.105)			
*R* ^2^	0.295			
Adjusted *R* ^2^	0.260			
Service rule commitment model				
Intercept		3.675^∗∗∗^ (0.053)	3.678^∗∗∗^ (0.052)	3.678^∗∗∗^ (0.050)
Tenure with supervisor		—	0.009 (0.013)	0.003 (0.013)
Age		—	0.015^∗^ (0.007)	0.015^∗^ (0.007)
Power distance		—	−0.173^∗^ (0.073)	−0.170^∗^ (0.072)
Team size		—	−0.002 (0.005)	−0.001 (0.005)
Servant leadership		—	—	0.178 (0.092)
Model statistics				
Random‐intercept variance, *τ*_00_		0.051	0.051	0.041
Level‐1 residual variance, *σ* ^2^		0.316	0.294	0.294
Pseudo‐*R* ^2^, Level 1		—	0.069	0.068
Pseudo‐*R* ^2^, Level 2		—	−0.009	0.188
Deviance		346.32	333.97	330.39

*Note:* For the service orientation model, unstandardized coefficients are reported with standard errors in parentheses; the model was estimated at the team level (*N* = 44 teams). For service rule commitment, unstandardized coefficients are reported with standard errors in parentheses. Model 0 is the unconditional model; Model 1 includes controls; and Model 2 adds servant leadership. Level‐1 controls and Level‐2 predictors were grand‐mean‐centered. Multilevel models were estimated using full maximum likelihood with random intercepts. *N* = 193 nurses nested within 44 teams.

^∗^ *p* < 0.05.

^∗∗∗^ *p* < 0.001.

#### 3.4.3. Multilevel Indirect Effects

Formal tests of the multilevel indirect effects are presented in Table 4. For the 2‐2‐1 pathway, servant leadership was positively associated with team service orientation (*a* = 0.428, *SE* = 0.105), and team service orientation was positively associated with nurses’ service performance (*b* = 0.371, *SE* = 0.149). The indirect effect through team service orientation was statistically significant (indirect effect = 0.159, Monte Carlo *SE* = 0.077, 95% CI [0.030, 0.328]), as the confidence interval did not include zero. For the 2‐1‐1 pathway, servant leadership was positively but not significantly associated with service rule commitment (*a* = 0.178, *SE* = 0.092). The between‐team component of service rule commitment was not significantly associated with nurses’ service performance (*b* = − 0.151, *SE* = 0.176). The indirect effect through service rule commitment was not statistically significant (indirect effect = − 0.027, Monte Carlo *SE* = 0.038, 95% CI [−0.115, 0.038]), as the confidence interval included zero. Accordingly, the proposed indirect pathway through team service orientation was supported, whereas the proposed indirect pathway through service rule commitment was not supported.

**TABLE 4:** Multilevel indirect effects linking servant leadership to nurses’ service performance.

Indirect pathway	a (SE)	b (SE)	Indirect effect	Monte Carlo SE	95% CI
Servant leadership ⟶ Team service orientation ⟶ Service performance	0.428 (0.105)	0.371 (0.149)	0.159	0.077	[0.030, 0.328]
Servant leadership ⟶ Service rule commitment ⟶ Service performance	0.178 (0.092)	−0.151 (0.176)	−0.027	0.038	[‐0.115, 0.038]

*Note:* Indirect effects were calculated using unstandardized coefficients. For the 2‐2‐1 pathway, a represents the association between servant leadership and team service orientation, and b represents the association between team service orientation and individual service performance. For the 2‐1‐1 pathway, b represents the between‐team association between the team‐mean component of service rule commitment and individual service performance. An indirect effect was considered statistically significant when the 95% Monte Carlo confidence interval did not include zero.

## 4. Discussion

This study examined the association between team‐level servant leadership and nurses’ service performance and tested two proposed multilevel indirect pathways through team service orientation and service rule commitment. Using a multilevel, time‐lagged, and multisource design, the findings showed that servant leadership was positively associated with nurses’ service performance. More importantly, the formal indirect‐effect tests showed a statistically significant indirect pathway through team service orientation, whereas the proposed indirect pathway through service rule commitment was not supported. These findings suggest that servant leadership may be more closely associated with the development of shared service values than with additional commitment to formal service rules.

The positive association between servant leadership and service performance extends previous research linking servant leadership to job satisfaction, organizational citizenship behavior, psychological safety, and performance‐related outcomes [6, 37]. By focusing on service performance, the present study moves beyond general attitudinal outcomes and addresses an outcome with direct relevance to nursing management and patient care. In clinical settings, service performance involves not only reliable and consistent task execution but also responsiveness, empathy, and patient‐centered interaction. The present findings therefore suggest that servant leadership may be relevant to the everyday service behaviors through which nurses contribute to the quality of patient care. This interpretation is also broadly consistent with evidence, showing that supervisor support is associated with employee performance and with work conditions that facilitate effective functioning [38]. A central contribution of the present study concerns the indirect pathway through team service orientation. Consistent with social learning theory [20], servant leaders may provide behavioral cues that emphasize humility, ethical responsibility, respect, and concern for others. Through repeated interaction with such leaders, nurses may come to share and enact service‐oriented values in their daily work. This interpretation is consistent with research in service settings, showing that servant leadership can influence employee attitudes and performance through identity‐based and motivational processes [15, 16, 39]. In nursing teams, service orientation may be particularly salient because it reflects a shared emphasis on attentive, responsive, and patient‐centered service. By contrast, the proposed indirect pathway through service rule commitment was not supported. Social exchange theory provides a basis for expecting employees who experience care, respect, and support from leaders to reciprocate through stronger commitment to organizational expectations. However, servant leadership is not primarily concerned with monitoring or enforcing formal rules. Its influence is more closely grounded in empowerment, ethical role modeling, support, humility, and concern for follower development. In professional nursing practice, where clinical standards and service expectations are already strongly institutionalized, commitment to formal service rules may function more as a basic role requirement than as an attitude that is substantially strengthened by leadership.

The contrast between the two pathways is theoretically informative. Team service orientation represents a value‐based pathway, whereas service rule commitment reflects adherence to formally prescribed service expectations. The statistically supported indirect pathway through team service orientation, together with the unsupported pathway through service rule commitment, suggests that servant leadership may be more closely aligned with the internalization and enactment of service values than with additional commitment to formal rules. Thus, value‐based and rule‐based pathways may differ in their relevance to understanding how servant leadership is associated with nurses’ service performance.

This interpretation nevertheless warrants caution. Previous studies have linked servant leadership to compliance‐oriented and extra‐role outcomes, including organizational citizenship behavior, procedural justice climate, and unit‐level performance [8, 37, 40]. The nonsignificant indirect pathway through service rule commitment in the present study should therefore not be interpreted as evidence that servant leadership is unrelated to rule‐consistent behavior. Rather, the findings suggest that the relative importance of value‐based and rule‐based pathways may vary across occupations and organizational contexts. In professional nursing settings, where clinical standards and service procedures are already embedded in routine practice, servant leadership may contribute more strongly by cultivating shared service values than by further strengthening commitment to formal service rules. Future research should examine whether this pattern is replicated across healthcare systems, cultural contexts, and different types of nursing units.

The study also makes a methodological contribution by adopting a multilevel, time‐lagged, and multisource design. Data were obtained from both nurses and supervisors, and the analyses explicitly accounted for the nesting of nurses within teams. This design reduces, although does not eliminate, concerns associated with common method bias and single‐source reporting. It also reflects the team‐based structure of nursing work, in which leadership may be experienced collectively, while service performance is enacted at the individual level. By formally distinguishing the 2‐2‐1 pathway through team service orientation from the 2‐1‐1 pathway through service rule commitment, the present study provides a more precise account of how team‐level leadership is associated with individual nurses’ service performance.

Overall, the findings underscore the relevance of value‐based leadership in nursing management. Servant leadership may help create a team context in which patient‐centered service values are observed, shared, and enacted. Rather than relying exclusively on formal service expectations, nurse managers may therefore benefit from modeling service‐oriented values and creating work environments that help nurses connect organizational standards with the meaning and practice of patient‐centered care.

## 5. Limitations

Several limitations should be acknowledged. First, although the study used a time‐lagged design, the two‐wave survey design does not establish definitive causality. The 4‐week interval provided temporal separation between nurses’ reports and supervisors’ ratings, but longitudinal designs with additional measurement occasions and experimental or quasiexperimental studies are needed to examine how servant leadership, service orientation, and service performance unfold over time. Second, common method bias cannot be completely ruled out. Although the time‐lagged and multisource design helped reduce this concern, servant leadership, service orientation, and service rule commitment were measured using nurses’ self‐reports. Future studies could incorporate peer ratings, patient‐reported care experiences, objective service quality indicators, or repeated longitudinal measurements to provide additional sources of evidence and further reduce this concern. Third, the sample was drawn from regional teaching hospitals in Taiwan, which may limit the generalizability of the findings to other healthcare systems, cultures, and care settings. Leadership expectations, service norms, and professional values may differ across countries and care contexts. Future studies should examine whether the proposed model applies to tertiary hospitals, long‐term care settings, community health services, and cross‐national samples. Fourth, this study examined only two proposed indirect pathways, through service orientation and service rule commitment. Other theoretically relevant factors, such as psychological empowerment, trust in leaders, moral identity, or team climate, may also help explain the association between servant leadership and service performance. Future research could test more comprehensive models that include these variables. Finally, the number of teams was relatively modest, which may have limited the precision and complexity of the multilevel analyses. The relatively modest ICC(2) for team service orientation also indicates limited reliability of the aggregated team mean. Accordingly, Level‐2 findings involving service orientation should be interpreted cautiously and replicated in future studies with larger numbers of teams and respondents per team. Larger studies would also permit the examination of more complex cross‐level models and additional contextual moderators, such as staffing adequacy and unit workload.

## 6. Conclusion

This study found that team‐level servant leadership was positively associated with nurses’ service performance. The findings further identified a statistically supported indirect pathway through team service orientation, whereas the proposed indirect pathway through service rule commitment was not supported. These results suggest that servant leadership may be more closely related to the internalization and enactment of patient‐centered values than to additional commitment to formal service rules. For nursing management, the findings highlight the importance of leadership practices that model humility, ethical responsibility, respect, and service to others. By fostering a service‐oriented team environment, servant leadership may help strengthen patient‐centered care and support consistent nursing service performance.

## 7. Implications for Nursing Management

The findings have several implications for nursing management. First, leadership development programs for head nurses and nurse managers should extend beyond administrative supervision and rule enforcement to emphasize value‐based leadership competencies. Case‐based workshops, reflective practice, mentoring, and peer coaching may help nurse managers strengthen humility, ethical decision‐making, empathic communication, respect for staff, and the role modeling of patient‐centered care. Second, hospitals can strengthen nurses’ service orientation by embedding servant leadership principles in everyday management practices. Nurse managers can use mentoring, case discussions, and team reflection to help nurses connect service standards with patient‐centered service values. Recognition systems may also highlight exemplary patient‐centered service behaviors, rather than focusing only on task completion or rule compliance. Third, nurse managers should create supportive work environments in which nurses feel respected and able to contribute to improvements in patient care. Equitable opportunities for professional development, transparent communication, and staff participation in decision‐making may help reinforce service orientation and support consistent service performance. Finally, the nonsignificant indirect pathway through service rule commitment suggests that formal service expectations may not fully explain how servant leadership is associated with nurses’ service performance. Nursing managers should therefore complement clear service standards with value‐based leadership practices that model and reinforce patient‐centered service values in everyday nursing practice.

## Author Contributions

Lihsiang Shih, Fenghuang Yang, and Lifen Wang contributed to the study conception and design. Lihsiang Shih, Fenghuang Yang, and Lifen Wang collected the data. Lifen Wang analyzed the data and drafted the manuscript.

## Funding

This research received no specific grant from any funding agency, whether public, commercial, or not‐for‐profit.

## Disclosure

All authors contributed to the interpretation of the findings, critically revised the manuscript, and approved the final version for submission.

## Conflicts of Interest

The authors declare no conflicts of interest.

## Data Availability

The data that support the findings of this study are available from the corresponding author upon reasonable request. The data are not publicly available due to privacy and ethical restrictions.
